# Multifunctional Plasmon-Tunable Au Nanostars and Their Applications in Highly Efficient Photothermal Inactivation and Ultra-Sensitive SERS Detection

**DOI:** 10.3390/nano12234232

**Published:** 2022-11-28

**Authors:** Tianxiang Zhou, Jie Huang, Wenshi Zhao, Rui Guo, Sicheng Cui, Yuqing Li, Xiaolong Zhang, Yang Liu, Qi Zhang

**Affiliations:** 1 Key Laboratory of Functional Materials Physics and Chemistry (Ministry of Education), College of Physics, Jilin Normal University, Changchun 130103, China; 2 Changchun Institute of Optics, Fine Mechanics and Physics, Chinese Academy of Sciences, Changchun 130033, China; 3 University of Chinese Academy of Sciences, Beijing 100049, China

**Keywords:** localized surface plasmon resonance, Au nanostars, photothermal therapy, surface-enhanced Raman scattering, FDTD

## Abstract

The development and application in different fields of multifunctional plasmonic nanoparticles (NPs) have always been research hotspots. Herein, multi-tip Au nanostars (NSs) with an anisotropic structure were fabricated for the photothermal therapy (PTT) of bacteria and surface-enhanced Raman scattering (SERS) detection of pollutants. The size and localized surface plasmon resonance (LSPR) characteristics of Au NSs were adjusted by varying Au seed additions. In addition, photothermal conversion performance of Au NSs with various Au seed additions was evaluated. Photothermal conversion efficiency of Au NSs with optimal Au seed additions (50 μL) was as high as 28.75% under 808 nm laser irradiation, and the heat generated was sufficient to kill *Staphylococcus aureus* (*S. aureus*). Importantly, Au NSs also exhibited excellent SERS activity for the 4-mercaptobenzoic acid (4-MBA) probe molecule, and the local electromagnetic field distribution of Au NSs was explored through finite-difference time-domain (FDTD) simulation. As verified by experiments, Au NSs’ SERS substrate could achieve a highly sensitive detection of a low concentration of potentially toxic pollutants such as methylene blue (MB) and bilirubin (BR). This work demonstrates a promising multifunctional nanoplatform with great potential for efficient photothermal inactivation and ultra-sensitive SERS detection.

## 1. Introduction

Recently, plasmonic nanoparticles (NPs) with localized surface plasmon resonance (LSPR) characteristics have attracted extensive interest due to their remarkable optical properties [1]. LSPR is caused by resonant interaction between free charge carriers and incident light, which can enhance absorption, scattering, and the electromagnetic field of particle surfaces [2,3,4,5]. Therefore, plasmonic NPs have been currently applied in various fields including photothermal therapy (PTT) [6,7,8], plasmon-driven photocatalytic environmental remediation [9], and surface-enhanced Raman scattering (SERS) detection [10,11,12,13,14]. Ag and Au are widely used because they are the few plasmonic materials that can support plasmon resonances in a visible range within the electromagnetic spectrum [15,16,17]. Although Ag NPs have a stronger LSPR effect due to their lower collision damping of electron plasma waves compared with Au NPs, the poor stability and severe toxicity in vivo of Ag NPs hinder their long-term practical applications [18,19,20]. In contrast, Au NPs have become the preferred plasmonic materials owing to their good chemical stability, excellent biocompatibility, high electrical conductivity, and high catalytic activity [21,22,23].

There is evidence suggesting that LSPR property depends strongly on the shape of plasmonic materials [24,25,26,27,28]. Compared with regular spherical nanocrystals, anisotropically shaped plasmonic NPs can not only tune LSPR in a certain wavelength range, but also exhibit greater field enhancement [29,30,31]. Based on the abovementioned reasons, the facile synthesis of the multi-tipped Au nanostars (NSs) with an anisotropic structure and their practical use in PTT and SERS detection have attracted more and more attention [32,33,34]. At present, the preparation methods of Au NSs mainly include the seed-mediated growth method [35], one-step synthesis method [36], and electron beam lithography [37]. The one-step synthesis method and electron beam lithography have inherent shortcomings compared with the seed-mediated growth method. For example, the one-step synthesis method has a simple preparation process; however, the competitive relationship between nucleation and seed growth may lead to the diversity of internal structures and the inhomogeneity of crystal size and shape [38,39]. Although electron beam lithography can precisely control the size and shape of particles, it is difficult to achieve practical applications due to its high cost and complex process [40,41,42,43]. At present, the seed-mediated growth method has been favored by researchers because of its high controllability, easy operation, low cost, and good reproducibility [38,44,45].

PTT coupled with SERS detection has been accepted as an effective and precise strategy for cancer theranostics [46]. PTT is a treatment method that transforms near-infrared (NIR) energy into hyperthermia, which can induce protein denaturation and cause irreversible damage to cells [47,48,49]. It has the characteristics of noninvasiveness, high selectivity, and causes little damage to normal tissue [50]. In addition, SERS, as a fast, lossless, and ultra-sensitive analytical technique, is widely used in analytical chemistry, biological sensing, and trace detection [51,52,53,54]. Fortunately, Au NSs can achieve the combination of PTT and SERS detection. Au NSs possess enhanced visible and NIR light absorption, which leads to higher absorption-to-scattering ratios compared with conventional phototherapy agents [55]. Au NSs also have high photothermal conversion efficiency, which is beneficial to generate a large quantity of heat to inactivate cancer cells [55,56,57]. For example, Yuan et al. realized the photothermal ablation of SKBR3 breast cancer cells using Au NSs as photothermal agents [58]. Furthermore, the experiments and theoretical simulations show that the multi-tipped Au NSs possess high-density “hot spots” generated by strong plasmonic coupling, which endows them with strong local electromagnetic field effects [12,59,60]. Therefore, Au NSs are also widely considered as a promising SERS detection platform [61,62]. For instance, Indrasekara et al. attached Au NSs to a thin Au film using 6-aminohexanethiol and achieved the femtomolar level detection of 4-mercaptobenzoic acid (4-MBA) [63]. Rodríguez-Lorenzo et al. reported a zeptomole detection limit of 1,5-naphtalenedithiol by sandwiching the molecule between an Au substrate and the tips of Au NSs [64]. However, many previous research studies have only concentrated on one aspect of PTT or SERS detection, and little attention has been carried out on the integration of PTT and SERS detection using colloidal Au NSs as both a phototherapy agent and a SERS substrate.

Inspired by the above ideas, we proposed and successfully prepared multi-tip Au NSs with an anisotropic structure through a seed-mediated growth method, which not only can realize the efficient photothermal inactivation of bacteria, but also can achieve the ultra-sensitive SERS detection of pollutants. Multifunctional Au NSs were selected as the nanoplatform for PTT inactivation, and the optimal photothermal conversion performance was obtained by adjusting Au seed additions. The photothermal inactivation ability of Au NSs with the optimum addition amount of Au seeds to *Staphylococcus aureus* (*S. aureus*) was investigated. Meanwhile, the signal enhancement capabilities of the multi-tipped Au NSs’ SERS active substrate were explored using 4-MBA as a reporter molecule. The finite-difference time-domain (FDTD) algorithm was applied to simulate the “hot spot” distribution of Au NSs, and a corresponding mechanism of SERS enhancement was analyzed. By measuring the SERS spectra of two kinds of pollutants, including methylene blue (MB) and bilirubin (BR) adsorbed on Au NSs, the universality of the application of Au NSs’ SERS substrate was estimated. This study explores the photothermal conversion mechanism and SERS enhancement mechanism of Au NSs and has great potential in the efficient inactivation of bacteria and ultra-sensitive detection of pollutants.

## 2. Experimental Section

### 2.1. Materials and Instruments

Details are listed in Supporting Information.

### 2.2. Fabrication of Au NSs

**Scheme 1:**
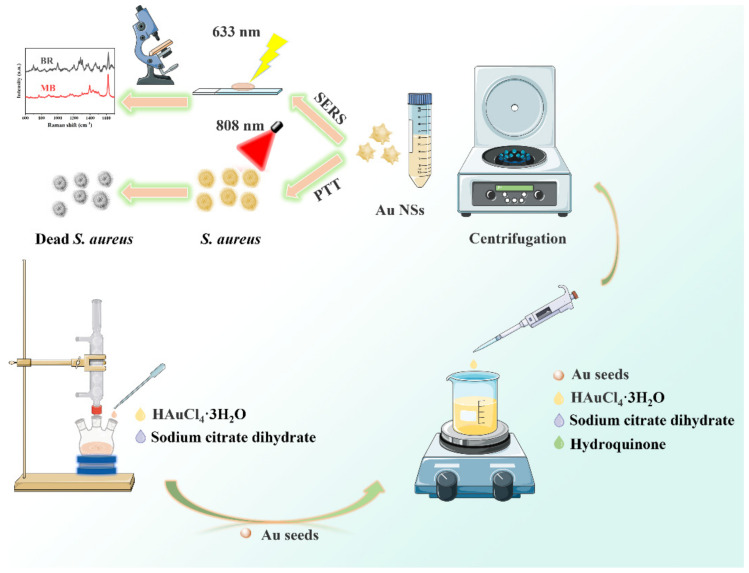
Schematic diagram of fabrication of Au NSs and their application in photothermal inactivation of bacteria and SERS detection of pollutants.

As shown in Scheme 1, the preparation of Au NSs could be divided into two steps, and the detailed preparation process could refer to previous research with minor modifications [45,46]. The first step was to prepare Au seeds using the citrate reduction method. Specifically, 27.95 mM HAuCl_4_·3H_2_O (1 mL) was injected into deionized water (99 mL) with magnetic stirring and heated to boiling. Next, 34 mM sodium citrate dihydrate (4 mL) was slowly dropped into the mixture and stirred uniformly until the mixture turned wine red. Au seeds were prepared after cooling the mixture. The second step was to synthesize Au NSs through seed-mediated growth method. Under magnetic stirring, 27.95 mM HAuCl_4_ 3H_2_O (150 µL) was dropped into deionized water (15 mL). Au seeds (150 µL), 34 mM sodium citrate dihydrate (33 µL), and 30 mM hydroquinone (1.5 mL) were then injected into the above solution. Mixed solution was centrifuged after stirring for 0.5 h, and Au NSs were obtained by washing three times using deionized water. The prepared Au NSs were placed in deionized water (3 mL) for later use.

### 2.3. Photothermal Conversion Performance of Au NSs

To study the influence of Au seed additions on the photothermal conversion performance and determine the best photothermal conversion agent (PCA), Au NSs with different Au seed additions were prepared. Au NSs with 50, 100, and 200 µL of Au seed additions were named A1, A2, and A3, respectively. Under an 808 nm laser at 1 W/cm^2^, A1, A2, and A3 were irradiated for 300 s. For comparison, a temperature change of deionized water under the same irradiation conditions was taken as the control group. After removing the laser, the solution was naturally cooled. During this process, temperature was monitored using an infrared (IR) thermal imaging camera at 50 s intervals.

### 2.4. PTT of S. aureus

To evaluate the photothermal sterilization ability of Au NSs in the NIR region, Au NSs were used to inactivate *S. aureus* under an 808 nm laser. A1 (100 μL) and *S. aureus* (10^5^ cfu/mL, 500 μL) were placed in well plates and incubated for 0.5 h at 37 °C. After irradiation using the 808 nm laser for 300 s, Calcein-AM/PI double staining kit was applied to explore survival rate of *S. aureus*. At the same time, three parallel experiments were performed, including *S. aureus* in presence or absence of laser therapy and *S. aureus* grown with A1 only. All fluorescence pictures were acquired using the MicroSpec System.

### 2.5. SERS Measurements and FDTD Algorithm Method

Details are listed in Supporting Information.

## 3. Results and Discussion

### 3.1. Fabrication and Characterization of Au NSs

**Figure 1:**
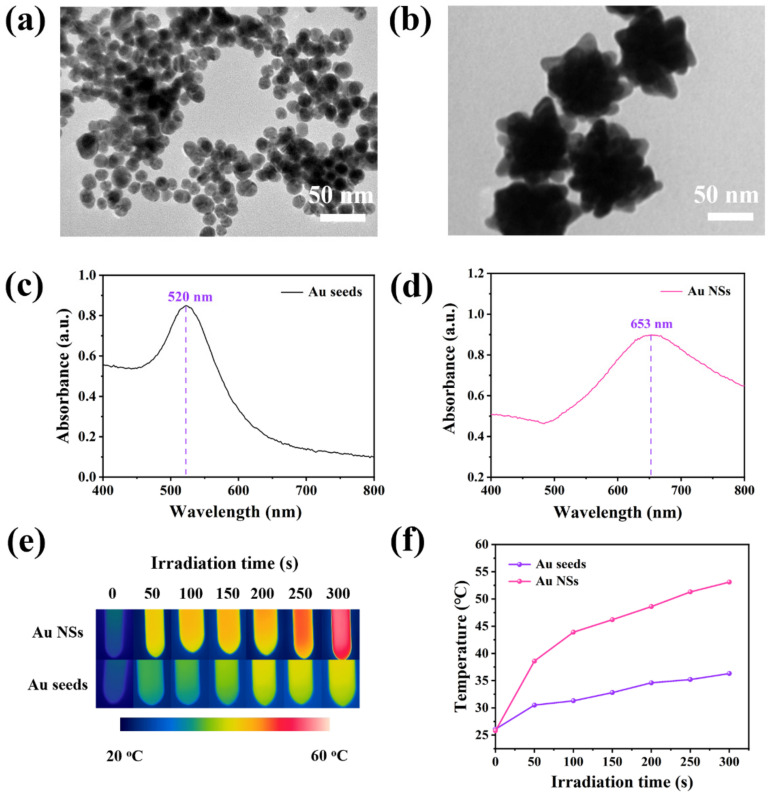
TEM images of (**a**) Au seeds and (**b**) Au NSs, UV-Vis spectra of (**c**) Au seeds and (**d**) Au NSs, (**e**) IR thermal images, and (**f**) corresponding time-dependent temperature curves of Au seeds and Au NSs.

TEM was used to observe morphologies of Au seeds and Au NSs. The average size of Au seeds is 13 nm, as illustrated in Figure 1a. The average size of Au NSs is about 92 nm in Figure 1b. Meanwhile, it can be observed that Au NSs are formed by a central core and many spikes. The irregular multi-branching morphologies of Au NSs are closely related to the synthesis steps. After HAuCl_4_·3H_2_O was reduced with sodium citrate dihydrate to obtain Au seeds, Au seeds were used as growth sites to spontaneously interact with Au NPs generated by a secondary reduction in HAuCl_4_·3H_2_O via hydroquinone, resulting in the formation of anisotropic Au NSs [65]. Figure 1c,d shows the UV-Vis spectra of Au seeds and Au NSs. Au seeds have a distinct LSPR peak at 520 nm, which is related to the longitudinal LSPR properties of Au NPs [66]. Au NSs have an LSPR absorption peak at 653 nm with a wider bandwidth and appear with a significant red-shift compared with Au seeds, which should be ascribed to the production of protruding tips based on the plasmon hybridization theory [66,67]. Especially, because the plasmon resonances of Au NSs are extended from the visible to NIR region, Au NSs have a better application prospect in PTT than spherical Au NPs [68]. Under 808 nm laser irradiation at 1 W/cm^2^, the photothermal performances of Au seeds and Au NSs were compared and the temperature changes were recorded by IR thermal images, as shown in Figure 1e. The temperature of Au seeds changes from 26.1 to 36.3 °C after irradiation for 300 s, which can be verified by color changes in IR thermal images (Figure 1e) and the corresponding time-dependent temperature curves (Figure 1f). In marked contrast, the temperature of Au NSs increases rapidly and reaches 53.1 °C under 808 nm laser irradiation. The results show that, under NIR laser irradiation, Au NSs can efficiently and quickly transform photon energy into heat and are expected to be ideal PCAs.

### 3.2. Effects of Different Au Seed Additions on Sizes and Optical Properties of Au NSs

**Figure 2:**
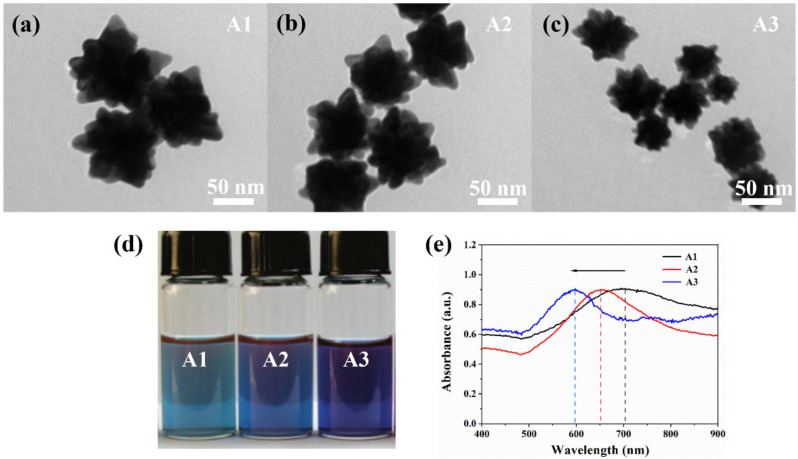
(**a**–**c**) TEM images, (**d**) corresponding solution color, and (**e**) UV-Vis spectra of A1, A2, and A3.

Given that the crystalline size of plasmonic nanometals is crucial for LSPR characteristics, the size of Au NSs was adjusted using various Au seed additions for determining optimal PCAs [69]. Morphologies of Au NSs with different Au seed additions were characterized by TEM (Figure 2a–c). The average crystalline size of A1 is close to 112 nm. The crystalline size of Au NSs decreases gradually with an increase in the amount of Au seeds. When the number of Au seeds is increased from 100 to 200 µL, the crystalline size of A2 and A3 decreases to 92.2 and 62.3 nm, respectively. The corresponding color change of Au NSs’ (A1, A2, and A3) colloid solution is shown in Figure 2d. The absorption spectra of the Au NSs (A1, A2, and A3) in Figure 2e show that the LSPR peaks of A1, A2, and A3 are located at 700, 653, and 600 nm, respectively. As expected, the optical absorption bands of multi-tip Au NSs show obvious size dependence. With an increase in the addition amount of Au seeds, LSPR peaks of Au NSs show blue shift. A possible explanation for this is that the excessive addition of Au seeds will cause the aggregation of the growth sites in the precursor solution, which hinders the further growth of Au NSs and thus leads to the blue shift of the absorption peaks [65].

### 3.3. Photothermal Effects of Au NSs and PTT of S. aureus by Au NSs

**Figure 3:**
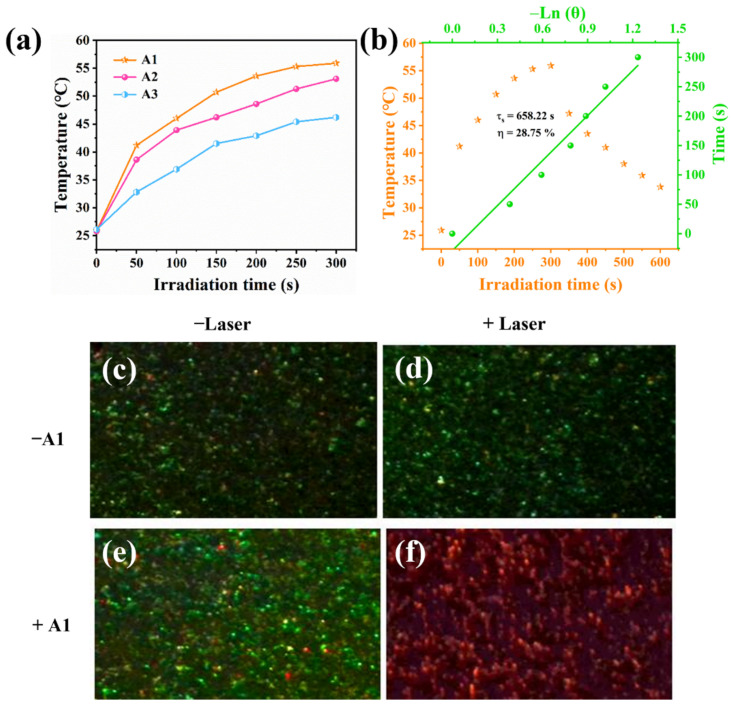
(**a**) Time-dependent temperature curves of A1, A2, and A3 under 808 nm laser irradiation at 1 W/cm^2^ for 300 s, (**b**) calculation of PCE of A1, live and dead fluorescence images of *S. aureus* treated with (**c**) none, (**d**) only NIR laser, (**e**) only A1, and (**f**) both A1 and NIR laser.

Considering the correlation between the LSPR position and photothermal effect, the photothermal conversion performance of A1, A2, and A3 was evaluated by recording their temperature changes [70]. The time-dependent temperature curves of A1, A2, and A3 irradiated with an 808 nm laser for 300 s are plotted in Figure 3a. It is observed that, with an increase in irradiation time, the temperature of the three samples increases and then tends to be stable. The temperature of A1, A2, and A3 increases to 55.9, 53.1, and 46.2 °C after 300 s, respectively. By comparison, the fastest rise in the temperature of A1 is observed at the same time, which may be due to the fact that the maximum value of the LSPR band of A1 is closest to the excitation wavelength [6,71]. For the purpose of quantifying the photothermal conversion capability of A1, photothermal conversion efficiency (PCE) was assessed via measuring the temperature changes of A1 under continuous irradiation with an 808 nm laser for 300 s. The PCE of A1 was 28.75%. The PCE of A1 in this work is higher than that in the previous work, which indicates that the appropriate LSPR regulation indeed has a positive significance in improving the photothermal conversion performance [72,73]. To investigate the plasmonic photothermal effect and cytotoxicity of Au NSs, an AM/PI double staining experiment was used to study the inactivation of A1 to *S. aureus* under 808 nm laser irradiation, where live bacteria appeared green and dead bacteria appeared red. *S. aureus* is still alive both with or without laser treatment, as shown in Figure 3c–f. The results show that laser irradiation alone cannot effectively inactivate bacteria, which directly confirm the noninvasive effect of laser irradiation on bacteria. In addition, almost no dead bacteria are found when *S. aureus* is treated with A1 alone, which indirectly indicates that the cytotoxicity of A1 to *S. aureus* is negligible. However, once *S. aureus* is treated with the A1 under laser irradiation, almost all of the *S. aureus* are killed. The possible bactericidal mechanism behind this is that the heat produced by Au NSs after NIR laser irradiation can cause the damage of membrane and denaturation of proteins and enzymes, which leads to bacterial death [74,75].

### 3.4. Mechanism of SERS Enhancement

**Figure 4:**
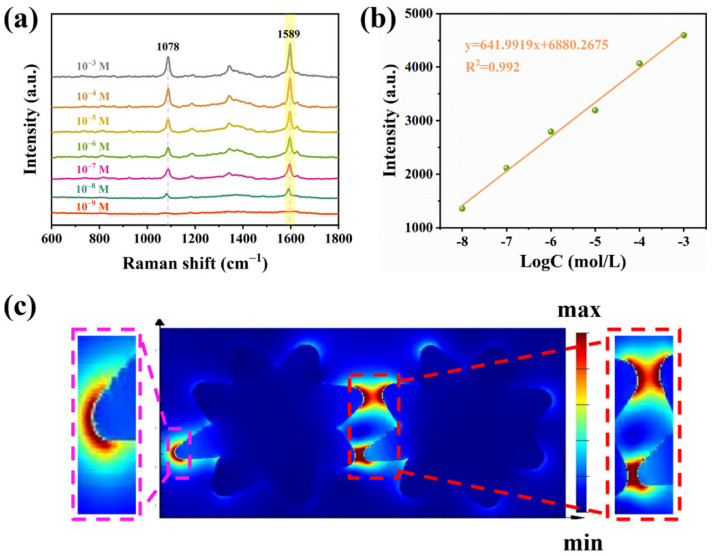
(**a**) SERS spectra of 4-MBA at various concentrations from 10^−3^ to 10^−9^ M, (**b**) corresponding plot of logarithmic concentration and SERS intensity of 4-MBA at 1589 cm^−1^, and (**c**) electric field distributions of adjacent A1.

The multi-tip Au NSs with an anisotropic structure in this work not only have excellent PTT ability, but also can be used as SERS substrates to realize rapid, non-destructive, and ultra-sensitive SERS detection. To explore the SERS detection capability of A1 SERS substrates, 4-MBA with thiol and carboxylic groups was selected as the reporter molecule. Figure 4a displays SERS spectra of 4-MBA at various concentrations from 10^−3^ to 10^−9^ M. SERS peak intensity of 4-MBA decreases with a decrease in concentration. The limit of detection (LOD) of 4-MBA is as low as 10^−8^ M because the characteristic peak can be distinguished even at a concentration of 10^−8^ M. Surprisingly, the LOD of A1 for 4-MBA in the study is significantly lower than the LOD of homogeneous Au nanostructures in previous research studies, indicating that multi-tip Au NSs have better SERS performance [76]. Moreover, Figure 4b exhibits the relationship between the concentrations of 4-MBA adsorbed on A1 and the corresponding SERS intensity at 1589 cm^−1^. The SERS intensity of 4-MBA shows a linear relationship with a logarithm of concentrations of 4-MBA and correlation coefficients (R^2^) which exceed 0.99, which implies that the A1 substrates can achieve quantitative SERS detection of the target reporter molecule. We further studied the SERS enhancement mechanism of 4-MBA adsorbed on A1. It is well known that the electromagnetic mechanism generated through the excitation of LSPR on noble metal nanostructures will greatly enhance the SERS performance, therefore it is recognized to be the main contribution of the SERS enhancement [77,78,79]. In fact, the electromagnetic enhancement (EM) mechanism mainly comes from the coupling interactions between adjacent noble metals, which produces a great electromagnetic field enhancement effect (so-called hot spot effect) in the nanogaps or near the nanotips [80,81,82,83]. The electromagnetic field intensity distribution of adjacent A1 under periodic boundary conditions was analyzed using the FDTD theoretical algorithm. As illustrated in Figure 4c, abundant hot spots are produced at spikes of A1 due to the “tip effect” [84,85,86]. More importantly, the hot spots also emerged near the region of the gap between adjacent A1, which will undoubtedly provide a strong SERS enhancement due to the inevitable aggregation of Au NSs in the actual detection process [87,88]. Therefore, plentiful hot spots ensure the excellent SERS detection effect of A1 SERS substrates.

### 3.5. Application of A1 in Detection of Different Types of Pollutants

**Figure 5:**
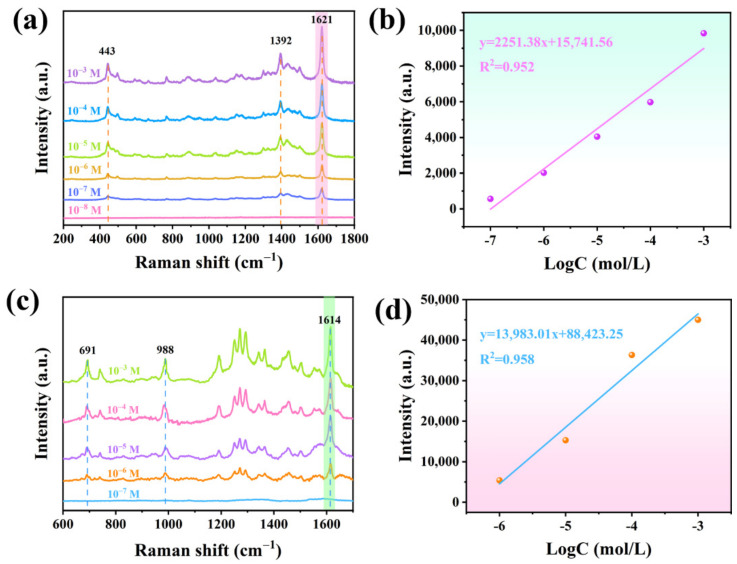
(**a**) SERS spectra of MB at various concentrations from 10^−3^ to 10^−8^ M and (**b**) the corresponding plot of SERS intensity and logarithmic concentration of MB at 1621 cm^−1^. (**c**) SERS spectra of BR at various concentrations from 10^−3^ to 10^−7^ M and (**d**) corresponding plot of logarithmic concentration and SERS intensity of BR at 1614 cm^−1^.

For the purpose of evaluating the universality of A1 SERS substrates, the SERS spectra of two kinds of pollutants including MB and BR adsorbed on A1 were measured, respectively. As a representative organic dye, MB is extensively used in different fields, such as textile printing, leather dyeing, coating manufacture, and food processing, due to its good solubility and color stability. However, if MB is released into the environment without proper treatment, it will cause great harm to the environment and to human health. Hence, a fast and sensitive method to detect MB is urgently needed [89,90]. Figure 5a presents the SERS spectra of MB at various concentrations (10^−3^–10^−8^ M), and the LOD of MB is as low as 10^−7^ M. The SERS intensity of MB at 1621 cm^−1^ has a linear relationship with a logarithm of concentrations, and the R^2^ is 0.952, as shown in Figure 5b. BR is a metabolite originated from the breakdown of red blood cells and other cells with porphyrins, which is the major pigment in human bile [91,92]. Unfortunately, unconjugated hyperbilirubinemia can result in an accumulation in BR in particular parts of the brain and cause permanent impairment to the brain and nervous system, especially for newborns [91,92]. Worst of all, a low level of BR can cause kidney dysfunction, therefore it is also crucial for the sensitive quantitative detection of BR. Figure 5c presents the SERS spectra of BR with various concentrations adsorbed on A1 SERS substrates, and the LOD of BR can be as low as 10^−6^ M. For further intuitive analysis of the relationship between SERS intensity and concentrations, Figure 5d depicts a corresponding plot of the logarithmic concentration of BR and SERS intensity. The peak at 1614 cm^−1^ is selected for linear fitting. Linear correlation coefficient is R^2^ = 0.958. The above results show that Au NSs can be applied to multi-purpose SERS substrates to achieve the sensitive detection of different types of pollutants.

## 4. Conclusions

In summary, multifunctional Au NSs for efficient bacterial photothermal inactivation and ultra-sensitive SERS detection of pollutants were successfully prepared. Multi-tip Au NSs with an anisotropic structure had better photothermal conversion performance by comparing the heating rates of Au seeds and Au NSs under NIR laser irradiation, which was related to the extension of the plasma resonance of Au NSs from a visible region to the NIR region. By changing the addition amount of Au seeds, the size and LSPR characteristics of Au NSs could be adjusted to realize the optimization of PCAs. Under the optimal condition, Au NSs showed high PCE (28.75%) during PTT, which could effectively inactivate *S. aureus*. Furthermore, 4-MBA was used as a probe molecule to evaluate the SERS performance of Au NSs’ SERS substrate, and the SERS enhancement mechanism was also discussed. The applicability of Au NSs as a SERS substrate was evaluated using the quantitative analysis of pollutants including MB and BR. When the concentrations of MB and BR were in the range of 10^−3^–10^−8^ and 10^−3^–10^−7^ M, the SERS signal intensity displayed a strong linear correlation with the logarithm of the concentrations of MB (y = 2251.38x + 15741.56, R^2^ = 0.952) and BR (y = 13983.01x + 88423.25, R^2^ = 0.958), and the LOD was as low as 10^−7^ and 10^−6^ M, respectively. This may be due to the presence of abundant hot spots at the tips of Au NSs and near the gap between adjacent Au NSs. This work provides a novel way to inactivate bacteria and has great potential in the detection of environmental pollutants.

## Data Availability

Not applicable.

## Supplementary Materials

The following supporting information can be downloaded at: https://www.mdpi.com/article/10.3390/nano12234232/s1, Materials, Instruments, SERS measurements, FDTD algorithm method, Photothermal conversion efficiency (PCE) of Au NSs. References [93,94,95,96] are cited in the Supplementary Materials.
