# Supplementary material for: Multifunctional Plasmon-Tunable Au Nanostars and Their Applications in Highly Efficient Photothermal Inactivation and Ultra-Sensitive SERS Detection
Source: Nanomaterials (Basel). 2022 Nov 28;12(23):4232. doi: 10.3390/nano12234232 (PMC9738658; doi:10.3390/nano12234232)
Supplement: Supplementary file 1 [file nanomaterials-12-04232-s001.zip › Figures.pdf]

Scheme S1.

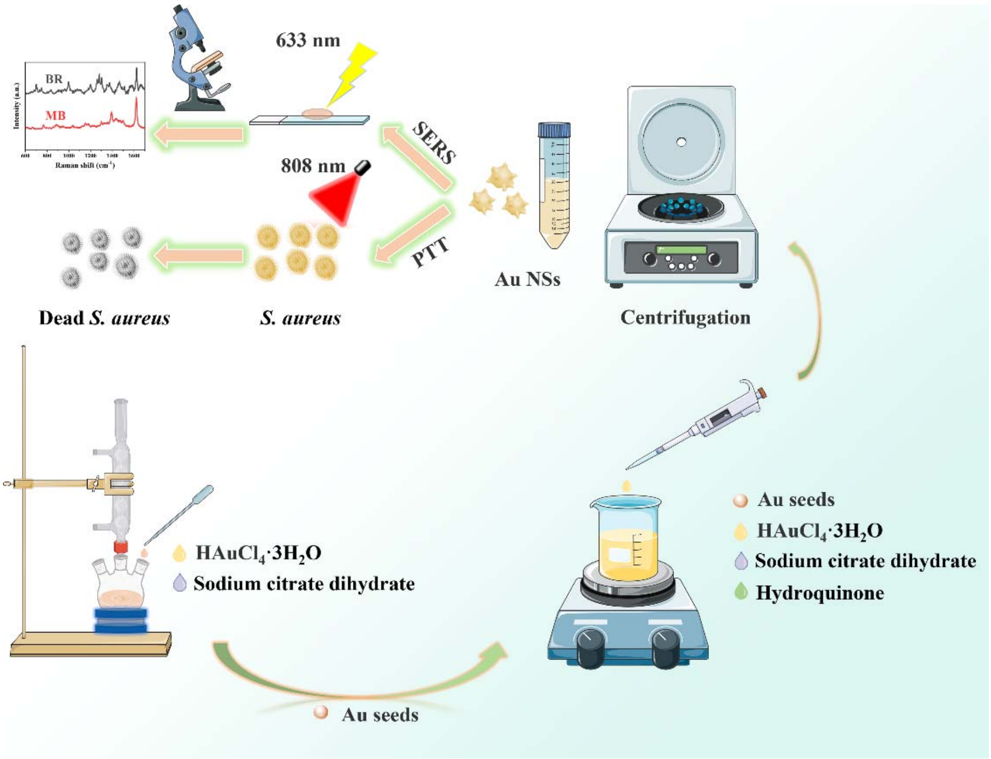

Scheme S1. Synthetic route for Au NSs and schematic diagram of photothermal inactivation of bacteria and SERS detection of pollutants.

Figure S1.

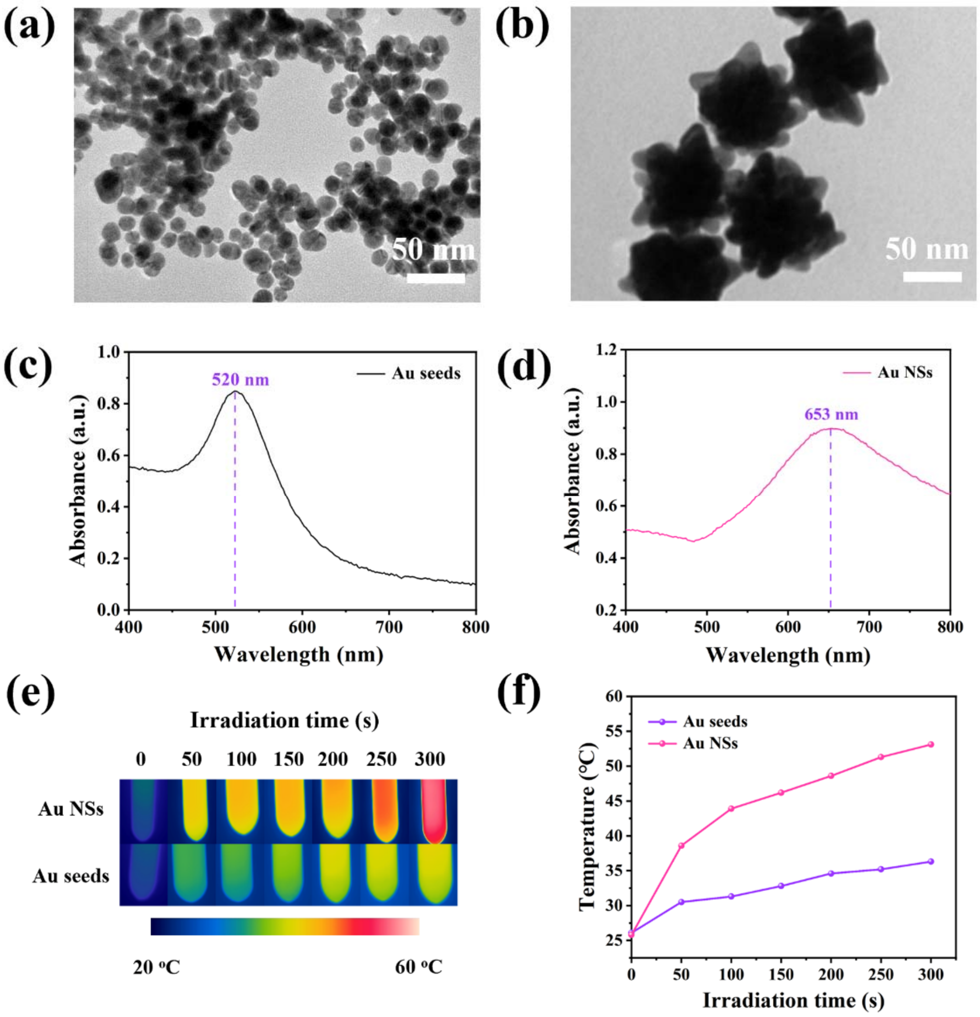

Figure S1. TEM images of (a) Au seeds and (b) Au NSs, UV-Vis spectra of (c) Au seeds and (d) Au NSs, (e) IR thermal images and (f) corresponding time-dependent temperature curves of Au seeds and Au NSs.

Figure S2.

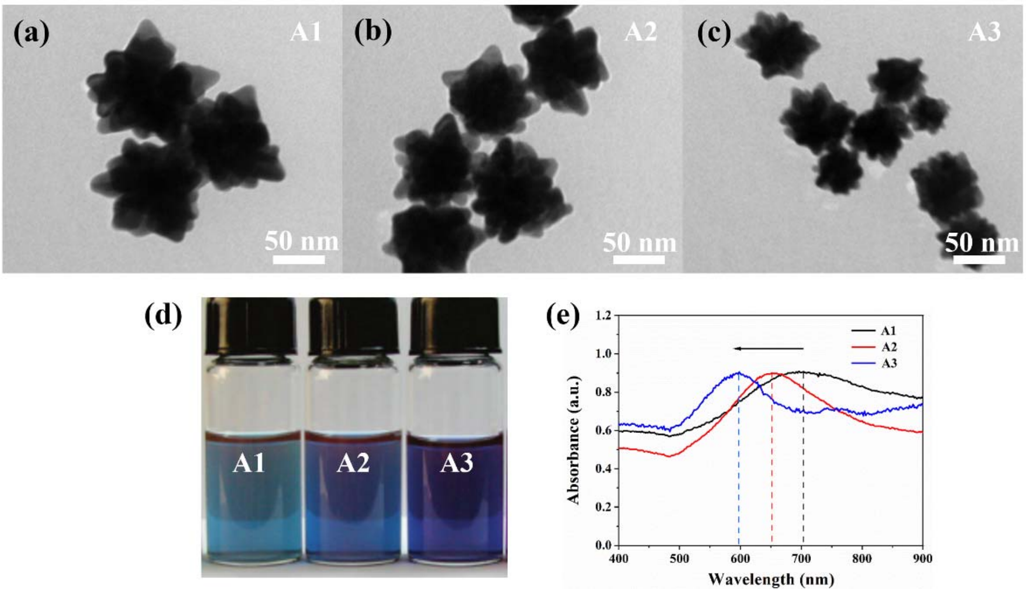

Figure S2. (a)-(c) TEM images, (d) corresponding solution color and (e) UV-Vis spectra of A1, A2 and A3.

Figure S3.

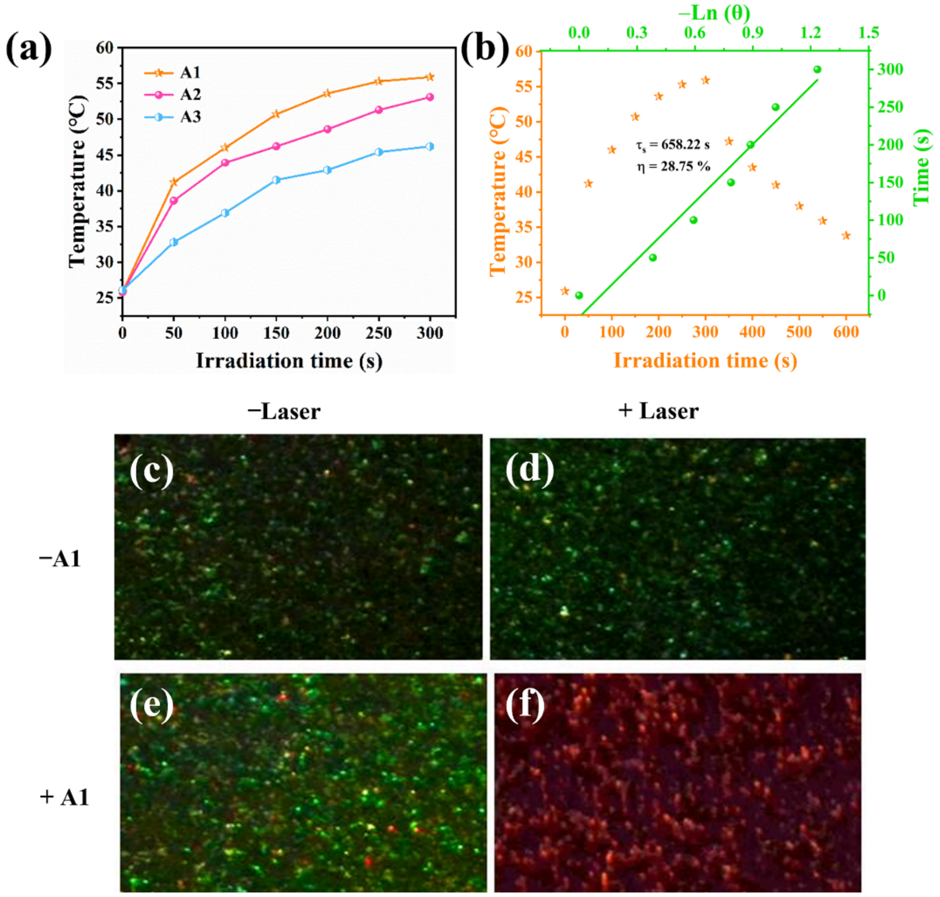

Figure S3. (a) Time-dependent temperature curves of A1, A2 and A3 under 808 nm laser irradiation at 1 W/cm^2 for 300 s, (b) calculation of η of A1, live and dead fluorescence images of *S. aureus* treated with (c) none, (d) only NIR laser, (e) only A1 and (f) both A1 and NIR laser.

Figure S4.

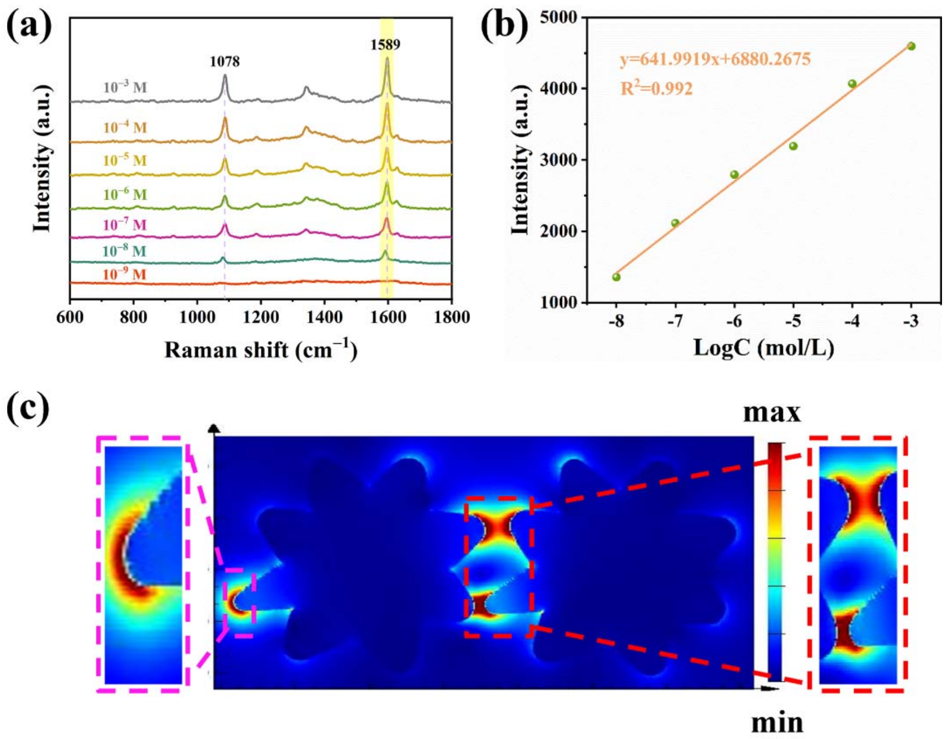

Figure S4. (a) SERS spectra of 4-MBA with different concentrations from 10^{-3} to 10^{-9} M, (b) the corresponding plot of SERS intensity and logarithmic concentration of 4-MBA at 1589 cm^{-1} and (c) electric field distributions of adjacent A1.

Figure S5.

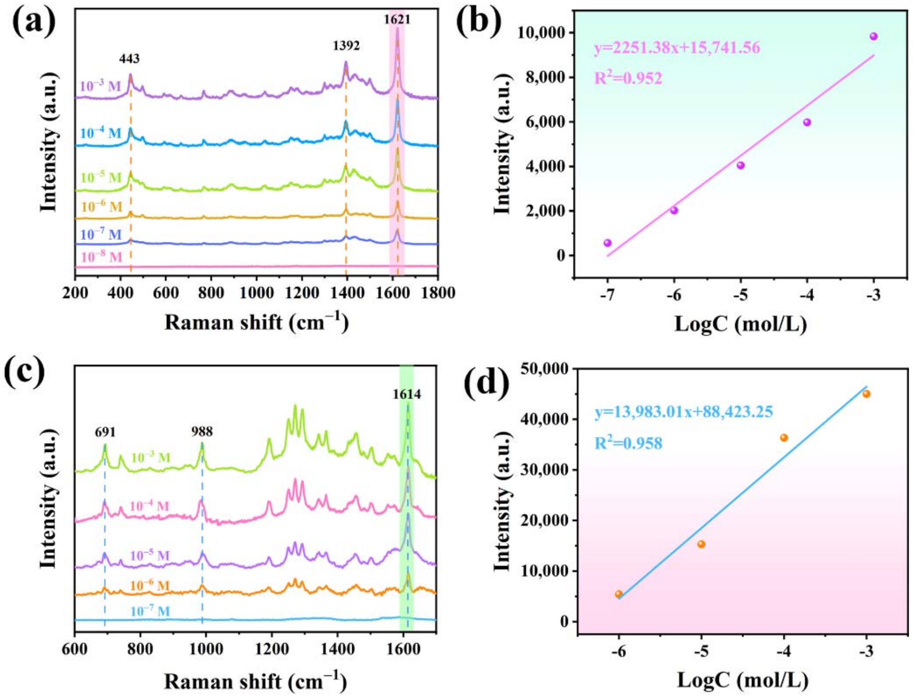

Figure S5. (a) SERS spectra of MB with different concentrations from 10^{-3} to 10^{-8} M and (b) the corresponding plot of SERS intensity and logarithmic concentration of MB at 1621 cm^{-1}. (c) SERS spectra of BR with different concentrations from 10^{-3} to 10^{-7} M and (d) the corresponding plot of SERS intensity and logarithmic concentration of BR at 1614 cm^{-1}.
