# Supplementary material for: Multifunctional Plasmon-Tunable Au Nanostars and Their Applications in Highly Efficient Photothermal Inactivation and Ultra-Sensitive SERS Detection
Source: Nanomaterials (Basel). 2022 Nov 28;12(23):4232. doi: 10.3390/nano12234232 (PMC9738658; doi:10.3390/nano12234232)
Supplement: Supplementary file 1 [file nanomaterials-12-04232-s001.zip › Highlights.pdf]

Highlights

- Mltifunctional Au nanostars (NSs) were designed and synthesized successfully.
- Au NSs had good photothermal effect and high SERS activity.
- Distribution of electromagnetic field of Au NSs was studied using FDTD method.
- Au NSs can achieve the efficient inactivation of *Staphylococcus aureus*.
- Detection limit of MB and BR by Au NSs was as low as 10^{-7} and 10^{-6} M, respectively.
