# Supplementary material for: Multifunctional Plasmon-Tunable Au Nanostars and Their Applications in Highly Efficient Photothermal Inactivation and Ultra-Sensitive SERS Detection
Source: Nanomaterials (Basel). 2022 Nov 28;12(23):4232. doi: 10.3390/nano12234232 (PMC9738658; doi:10.3390/nano12234232)
Supplement: Supplementary file 1 [file nanomaterials-12-04232-s001.zip › Supporting Information_nanomaterials-2037023.pdf]

Multifunctional plasmon-tunable Au nanostars and their applications in highly efficient photothermal inactivation and ultra-sensitive SERS detection

Tianxiang Zhou [1], Jie Huang [1], Wenshi Zhao [1,2,3], Rui Guo [1], Sicheng Cui [1], Yuqing Li [1], Xiaolong Zhang [1], Yang Liu [1,*] and Qi Zhang [1,*]

[1] Key Laboratory of Functional Materials Physics and Chemistry (Ministry of Education), College of Physics, Jilin Normal University, Changchun 130103, China

[2] Changchun Institute of Optics, Fine Mechanics and Physics, Chinese Academy of Sciences, Changchun 130033, China

[3] University of Chinese Academy of Sciences, Beijing 100049, China

* Correspondence: liuyang@jlnu.edu.cn (Y.L.); qzh7512@126.com (Q.Z.).

Materials

Gold (III) chloride trihydrate ($HAuCl_4 \cdot 3H_2O$) and methylene blue (MB) were procured from Sinopharm Chemical Reagent Co., Ltd. Sodium citrate dihydrate ($Na_3C_6H_5O_7 \cdot 2H_2O$), hydroquinone (98%), bilirubin (>98%) and 4-mercaptobenzoic acid (4-MBA) were ordered from Aladdin Reagent Co., Ltd. Calcein-AM/PI Double Stain Kit was purchased from Shanghai Yeasen Biotech Co., Ltd. *Staphylococcus aureus* (*S. aureus*) shock-frozen strains were obtained from MiaoLing Plasmid Sharing Platform. Deionized water purified with Milli-Q system (18.2 MΩ cm^{-1}) was utilized through whole experiment. All aforementioned chemical reagents were used as received.

Instruments

The morphologies of samples were characterized by transmission electron microscope (TEM; JEOL 2100). Ultraviolet-visible (UV-Vis) spectra were measured with a Shimadzu UV 3600 spectrophotometer (Shimadzu Corporation, Japan). SERS spectra were collected by a Renishaw inVia Raman spectrometer (Renishaw, London, UK). Live-dead assays of bacteria were investigated by MicroSpec (FORTEC, Hong Kong). Temperature increase experiments were induced using 808 nm multi-mode laser diode (LR-MFJ-808/2000 mW, China) and an infrared thermal imaging camera (FOTRIC 326C-L44, China) was used to record the temperature.

SERS measurements

In this work, 100 μL of A1 was added into 500 μL of different concentrations of 4-MBA solution (10^{-3} to 10^{-9} M), respectively. Then, the mixture was blended and shaken on an oscillator without light condition for several minutes. The obtained samples were dropped on the aluminum pan for SERS detection. SERS spectroscopy was performed at 633 nm laser with an energy density of 10 mW. The SERS spectra of the system were scanned from 600 to 1800 cm^{-1} and acquisition time was about 10 s. MB and BR were the same as the SERS detection process of the above detection process of 4-MBA.

FDTD algorithm method

The electromagnetic field distribution of A1 was simulated by using FDTD

software (Lumerical solutions). The simulation region was an 500 × 500 × 500 nm^3 cuboid space surrounded by absorber layers to avoid numerical reflections. And the structural parameters in the FDTD simulation were similar to the observed values of the A1 in the experiment. A continuous-wave source (λ= 633 nm) was placed in the positive z-axis. The polarization direction of the source was along the y-axis.

Photothermal conversion efficiency (PCE) of Au NSs

The value of PCE was calculated according to the previous literature by the following equation (1): [1-4]

$$\eta = \frac{hS(T_{max} - T_{surr}) - Q_{dis}}{I(1 - 10^{-A_\lambda})} \tag{1}$$

where T_{max} is the maximum equilibrium temperature of solution (55.9 °C), T_{surr} is the surrounding ambient temperature (25.9 °C), I is the power of 808 nm laser (0.785 W, 1.0 W/cm^2, spot diameter = 10 mm) and A_λ is the absorbance of A1 at 808 nm (0.819). Q_{dis} represents the heat dissipation from light absorption by the solvent and the container and it is so small that it can be negligible. The cooling process of A1 was also monitored and the value of hS was calculated using the following equation (2):

$$hS = \frac{\sum mC_p}{\tau_s} \tag{2}$$

where m and C_p are the mass (1 g) and heat capacity (4.2 J/(g·°C)) of the solvent, respectively. τ_s is the time constant of heat transfer of the system and is an unknown value. In order to obtain this unknown quantity, a dimensionless quantity θ need to be defined. τ_s is determined by linear fitting using the cooling times of the natural cooling stage (between 300 s and 600 s) and the negative natural logarithm of θ, as shown in Figure 3b. Equations (3) and (4) were introduced:

$$t = -\tau_s \ln \theta \tag{3}$$

$$\theta = \frac{T - T_{surr}}{T_{max} - T_{surr}} \tag{4}$$

where t is the cooling time after irradiation, T represents the solution temperature and θ is called driving force temperature. After substituting the corresponding values into the equations (2), (3), (4) and performing linear fitting, it is obtained that τ_s is the slope value (658.22 s) and hS is 6.38 mW/°C. Subsequently, the values acquired from the above equations and the remaining parameter values are substituted into equation (1).

Therefore, the PCE of A1 can be calculated to be 28.75%.

1. Ji, M.; Xu, M.; Zhang, W.; Yang, Z.; Huang, L.; Liu, J.; Zhang, Y.; Gu, L.; Yu, Y.; Hao, W.; An, P.; Zheng, L.; Zhu, H.; Zhang, J. Structurally Well-Defined Au@$Cu_{2-x}S$ Core-Shell Nanocrystals for Improved Cancer Treatment Based on Enhanced Photothermal Efficiency. *Adv. Mater.* **2016**, 28, 3094-3101.
2. Chen, X.; Zhang, M.; Li, S.; Li, L.; Zhang, L.; Wang, T.; Yu, M.; Mou, Z.; Wang, C. Facile synthesis of polypyrrole@metal-organic framework core-shell nanocomposites for dual-mode imaging and synergistic chemo-photothermal therapy of cancer cells. *J. Mater. Chem. B* **2017**, 5, 1772-1778.
3. Yu, Y.; Chi, B.; Lin, L.; Yang, Z.; He, Q.; Xu, Z.; Yi, C.; Wang, J. Microwave-assisted preparation of paramagnetic zwitterionic amphiphilic copolymer hybrid molybdenum disulfide for T1-weighted magnetic resonance imaging-guided photothermal therapy. *J. Mater. Chem. B* **2018**, 6, 6391-6398.
4. Chen, Z.; Xia, Q.; Zhou, Y.; Li, X.; Qi, L.; Feng, Q.; Liu, R.; Chen, W. 2-Dicyanomethylenethiazole based NIR absorbing organic nanoparticles for photothermal therapy and photoacoustic imaging. *J. Mater. Chem. B* **2019**, 7, 3950-3957.
